# Benzo(a)pyrene Enhanced Dermatophagoides Group 1 (Der f 1)-Induced TGFβ1 Signaling Activation Through the Aryl Hydrocarbon Receptor–RhoA Axis in Asthma

**DOI:** 10.3389/fimmu.2021.643260

**Published:** 2021-04-15

**Authors:** Eryi Wang, Wei Tu, Danh C. Do, Xiaojun Xiao, Shehar B. Bhatti, Liteng Yang, Xizhuo Sun, Damo Xu, Pingchang Yang, Shau-Ku Huang, Peisong Gao, Zhigang Liu

**Affiliations:** ^1^Department of Respiratory and Allergy, Third Affiliated Hospital of Shenzhen University, Shenzhen, China; ^2^The State Key Laboratory of Respiratory Disease for Allergy, Shenzhen Key Laboratory of Allergy and Immunology, Shenzhen University School of Medicine, Shenzhen, China; ^3^Johns Hopkins Asthma and Allergy Center, Johns Hopkins University School of Medicine, Baltimore, MD, United States; ^4^National Institute of Environmental Health Sciences, National Health Research Institutes, Miaoli, Taiwan

**Keywords:** RhoA, TGFβ1, BaP (6-benzylaminopurine), Der f 1, aryl hydrocarbon receptor

## Abstract

We have previously demonstrated that benzo(a)pyrene (BaP) co-exposure with dermatophagoides group 1 allergen (Der f 1) can potentiate Der f 1-induced airway inflammation. The underlying mechanism, however, remains undetermined. Here we investigated the molecular mechanisms underlying the potentiation of BaP exposure on Der f 1-induced airway inflammation in asthma. We found that BaP co-exposure potentiated Der f 1-induced TGFβ1 secretion and signaling activation in human bronchial epithelial cells (HBECs) and the airways of asthma mouse model. Moreover, BaP exposure alone or co-exposure with Der f 1-induced aryl hydrocarbon receptor (AhR) activity was determined by using an AhR-dioxin-responsive element reporter plasmid. The BaP and Der f 1 co-exposure-induced TGFβ1 expression and signaling activation were attenuated by either AhR antagonist CH223191 or AhR knockdown in HBECs. Furthermore, AhR knockdown led to the reduction of BaP and Der f 1 co-exposure-induced active RhoA. Inhibition of RhoA signaling with fasudil, a RhoA/ROCK inhibitor, suppressed BaP and Der f 1 co-exposure-induced TGFβ1 expression and signaling activation. This was further confirmed in HBECs expressing constitutively active RhoA (RhoA-L63) or dominant-negative RhoA (RhoA-N19). Luciferase reporter assays showed prominently increased promoter activities for the AhR binding sites in the promoter region of RhoA. Inhibition of RhoA suppressed BaP and Der f 1 co-exposure-induced airway hyper-responsiveness, Th2-associated airway inflammation, and TGFβ1 signaling activation in asthma. Our studies reveal a previously unidentified functional axis of AhR–RhoA in regulating TGFβ1 expression and signaling activation, representing a potential therapeutic target for allergic asthma.

## Introduction

Air pollution, especially exposure to particulate matter, is one of the major risk factors for the increased prevalence of childhood asthma worldwide (1). High levels of particulate matter [i.e., diesel exhaust particles (DEP)] can enhance the risk of atopic sensitization and exacerbation of asthma (2–4). Especially prenatal exposure to DEP or DEP-derived polycyclic aromatic hydrocarbons (PAHs) is associated with atopic sensitization, early childhood wheeze, and asthma (5, 6). Most importantly, DEP co-exposure with house dust mite can exacerbate allergic sensitization and induce key features of asthma mouse models (7–11). So far, many epidemiological and clinical studies have suggested a significant link between exposure to environmental pollutants and allergens and allergic airway inflammation and asthma. However, the causal relationship and underlying molecular mechanisms are poorly characterized.

Benzo(a)pyrene (BaP) is one of a large number of PAHs that are formed during the incomplete combustion of organic matter (12). Mixtures of PAHs including BaP are ubiquitous in the air because they are generally derived from cigarettes, barbecue grills, automobile exhausts, and industrial combustion (12, 13). Exposure to BaP alone has been shown to induce oxidative stress, bronchial epithelium injury, and inflammation (14–16). Mechanistically, BaP can directly activate aryl hydrocarbon receptor (AhR), induce IL-33 expression and eosinophil infiltration in a mouse model of allergic airway inflammation (17), and elicit T-helper 2-driven pro-inflammatory responses in a mouse model of allergic dermatitis (18). AhR, as a ligand-activated transcription factor, can be activated by small molecules in various diets, metabolites, microorganisms, and pollutants (19–24) and plays an important role in the regulation of innate and adaptive immune responses (25–28). Of note is that we have recently made a novel finding that BaP co-exposure with dermatophagoides farina group 1 allergen (Der f 1) can potentiate Der f 1-induced airway hyper-responsiveness (AHR) and lung inflammation (29). Particularly, BaP co-exposure can promote Der f 1-induced oxidative stress (ROS) and IL-25, IL-33, and TSLP release from airway epithelial cells. Furthermore, BaP exposure-activated AhR can promote Der f 1-induced oxidative stress and epithelial cytokine release that contribute to enhanced allergic airway inflammation. However, the relationship between BaP-activated AhR and airway inflammation is poorly known.

Transforming growth factor β1 (TGFβ1) is a pleiotropic regulator of immune responses and plays an important role in cell growth, differentiation, migration, and activation depending on the environmental trigger, cell type, and microenvironment (30–35). Airway epithelial cells are a major source of pulmonary TGFβ1, and activation of TGFβ1 signaling is essential in allergen-induced exacerbation of AHR and airway inflammation in asthma (34, 36). Thus, it is critical to determine the molecular mechanisms regarding the regulation of TGFβ1 signaling, which may provide novel insights into the mechanisms that lead to allergic airway inflammation. The ras homolog family member A (RhoA) of the Rho family GTPases has been considered to be one of the most promising and novel therapeutic targets for asthma (37). Studies including ours have suggested a positive loop between RhoA/Rho-kinase signaling and TGFβ1 that drives airway constriction, airway hyper-responsiveness, and airway remodeling in asthma (38–41), raising the possibility that RhoA/Rho-kinase signaling may be one of the central pathways in regulating allergen-induced TGFβ1 activation.

In the current study, we demonstrate that BaP co-exposure enhances Der f 1-induced TGFβ1expression and signaling activation in airway epithelial cells and the airways of asthma mouse model. Furthermore, BaP-activated AhR plays a critical role in Der f 1-induced TGFβ1 expression and signaling activation. Importantly, we made novel findings that BaP-activated AhR can bind to the promoter region of RhoA and regulate RhoA/Rho-kinase activation, and inhibition of RhoA significantly suppresses co-exposure-induced AHR, Th2-associated airway inflammation, and TGFβ1 signaling in a mouse model of asthma.

## Materials and Methods

### Animals

The experimental protocols in this study were reviewed and approved by the Animal Care and Use Committee in Peking University Shenzhen Graduate School and was in accordance with the guidelines and regulations of the institution. Both male and female C57BL/6 mice aged 6–8 weeks were purchased from the experimental animal center of Guangdong province. The animals were maintained under specific pathogen-free conditions at the animal facility of Shenzhen University.

### BaP Co-Exposure With Der f 1-Induced Mouse Model of Asthma

The generation of an asthma mouse model was established as previously described (29). Briefly, both male and female mice were sensitized and challenged every other week for 6 weeks with intranasal administrations of 25 μg Der f 1 (Indoor Biotechnologies) under isoflurane anesthesia. BaP (Sigma-Aldrich) was dissolved in borate saline buffer (Sigma-Aldrich) and intranasally administered at a concentration of 20 μM once every week during Der f 1 sensitization and challenge. In some cases, the mice were pre-treated with Fasudil or Y-27632 dissolved in 0.5% DMSO in phosphate-buffered saline (PBS) at a dose of 30 mg/ml by intraperitoneal administration 1 h before every single BaP treatment. Age- and gender-matched control mice were treated with PBS.

### Measurement of Airway Hyper-Responsiveness

Airway hyper-responsiveness (AHR) was measured 24 h after the last challenge with whole-body plethysmography (Buxco Europe Ltd., Winchester, UK) as previously described (29). Briefly, the mice were exposed to increasing doses of methacholine (Mch) (Sigma-Aldrich, St. Louis, USA) at 6.25, 12.5, 25, 50, and 100 mg/ml or PBS. Tests at two different concentrations were temporally separated to allow the respiratory intensity to drop back to baseline. The percentage curves for Penh values at different Mch doses were plotted, starting with PBS stimulation.

### Broncheoalveolar Lavage

Mice were sacrificed, and bronchoalveolar lavage (BAL) was performed by instillation of 0.8 ml of PBS through a tracheal cannula. BAL samples were centrifuged at 1,500 rpm for 5 min at 4°C. The supernatants were collected and stored at −80°C for cytokine analysis, and the cells were stained for the analysis of cellular compositions by Wright–Giemsa staining on microscope slides. The total number of eosinophils, neutrophils, and macrophages was determined by counting 200 leukocytes in randomly selected areas of the slides under light microscopy.

### Lung Pathology

The whole lung was fixed in 4% formalin for 24 h and embedded in paraffin after dehydration in alcohol. Lung sections (3 μM) were stained with hematoxylin and eosin (H&E) and periodic acid–Schiff (PAS) solution for histopathological analysis following the protocols as previously described (29).

### Cytokine Determination

IL-4, IL-5, IL-13, IFNγ, IL-17A, IL-25, IL-33, TSLP, and TGFβ1 in bronchoalveolar lavage fluid were measured by ELISA (Sizhengbo Inc., Beijing, China) according to the manufacturer's instructions.

### Der f 1-Specific IgE, IgG1, and IgG2α Detection

The levels of Der f 1-specific IgE, IgG1, and IgG2α in serum samples were determined by a standard ELISA as previously described (29).

### Immunofluorescence Staining

Sectioned lung tissues were first blocked using 5% w/v bovine serum albumin for 1 h, followed by incubation with the primary antibodies against Smad3 (EP568Y, Abcam), phospho-Smad3 (ab52903; Abcam), TGFβ1 (Ab179695; Abcam), RhoA-GTPase (26904; New East Biosciences), AhR (Ab84833; Abcam), and EpCAM (G8.8; ThermoFisher), respectively, overnight at 4°C. The sample sections were then incubated with secondary antibodies conjugated with Alexa Fluor dyes (ThermoFisher) at room temperature for 1 h. Isotype-matched negative control antibodies (R&D Systems) were used under the same conditions. The nuclei were counterstained with 6-diamidino-2-phenylindole, dihydrochloride (Solarbio, Beijing, China). Sections were mounted with the ProLong Gold Anti-fade Kit (Molecular Probes) and observed with a Nikon Eclipse Ti-U microscope equipped with a DS-Fi2 camera (Nikon). To determine the fluorescence signal in tissue sections, fluorescent-positive cells in four different high-power fields (500 μM^2^) from each slide were quantified using ImageJ v1.50e (National Institutes of Health). At least two tissue sections per mouse were included for analysis and presented as mean fluorescence intensity per square micrometer.

### Western Blotting

Western blotting was performed as previously described (29). Briefly, the collected cells were lysed in RIPA buffer (Sigma-Aldrich) containing protease and phosphatase inhibitor cocktails (Solarbio Inc., Beijing China). Protein concentration was measured using a bicinchoninic acid protein assay kit (Pierce). Aliquots of 30–50 μg protein samples were used for SDS-PAGE and then transferred to a polyvinylidene difluoride membrane (Invitrogen). After blocking with 5% non-fat milk in Tris-buffered saline with Tween-20 (TBST), the membrane was incubated with anti-RhoA GTPase (New East Bioscience, PA), AhR (Abcam), phosphor-Smad3 (Abcam), and total Smad3 (Abcam) or anti-β-actin antibodies. Detailed information is included in the online repository (Supplementary Table 1). Blots were visualized with a horseradish peroxidase-conjugated secondary antibody (Sizhengbo Inc., Beijing, China) and enhanced chemiluminescence Western blotting detection system (GE Life Sciences). Relative protein expression was determined by densitometric analysis using ImageJ (NIH).

### Cell Culture and Transfection

Human bronchial epithelial cells (16HBECs) were purchased from Haoge biological company (Shanghai, China) and cultured in Dulbecco's modified Eagle's medium, supplemented with 10% fetal bovine serum and 1% penicillin-streptomycin. The cells were maintained at 37°C in a humidified atmosphere at 5% CO_2_. 16HBECs were transfected with a plasmid expressing a constitutively active RhoA (RhoA-L63) or a dominant-negative RhoA (RhoA-N19) or empty vector as previously described (38). The efficiency of transfection was determined by Western blotting.

In a separate experiment, 16HBECs were seeded onto Nunc® Lab-Tek® II chamber slides (Millipore-Sigma) overnight and treated with either media alone, BaP, Der f1, or BaP–Der f1 combination. The cells were then fixed with 4% formalin, permeabilized with TBST, and stained for phospho-Smad3 and TGFβ1, and fluorescent signals were quantified as described above.

### AhR Activity Determination

**Figure 1:**
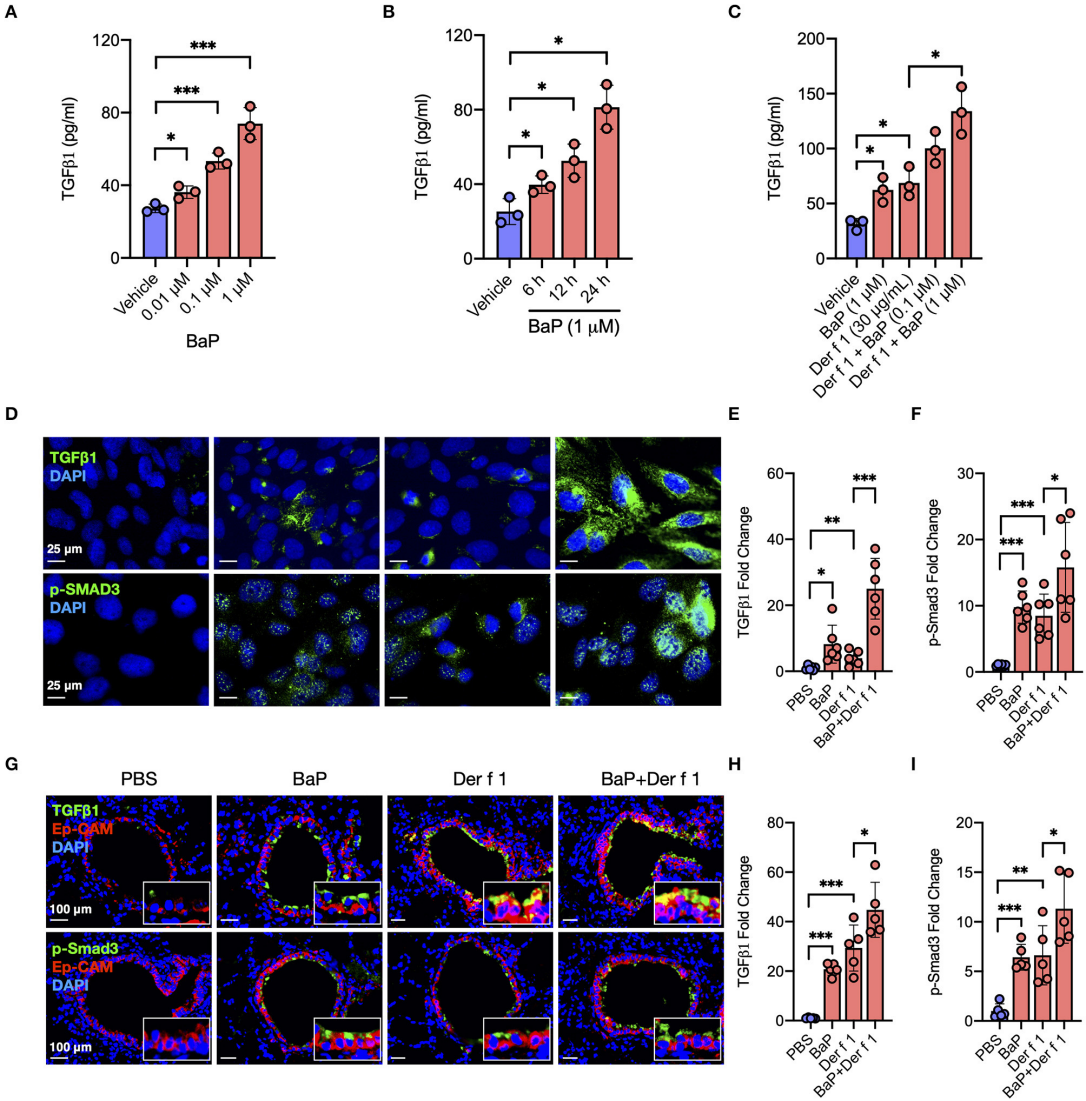
BaP co-exposure exacerbates Der f 1-induced TGFβ1 secretion and signaling activation. **(A,B)** Levels of TGFβ1 in supernatants of 16HBECs treated with BaP at varying doses (0.01–1 μM) **(A)** and different times (6–24 h) **(B)**. **(C)** Levels of TGFβ1 in supernatants of 16HBECs treated with BaP (1 μM) and Der f 1 (30 μg/ml) alone or in combination. **(D)** Expression of TGFβ1 and p-SMAD3 in the surface of 16HBECs treated with BaP (1 μM) and Der f 1 (30 μg/ml) alone or in combination. **(E,F)** Quantitative analysis of TGFβ1 **(E)** and p-Smad3 **(F)** fluorescent signal (*n* = 6). **(G)** Representative immunofluorescence images of TGFβ1 (green) and p-SMAD3 (green) expression in the airway epithelial cells (EpCAM, red) of an asthma mouse model. **(E,F)** Quantitative analysis of AhR and RhoA fluorescent signal in **(G)** (*n* = 6 mice/group). **(H,I)** Fluorescent-positive cells of tissue sections were quantified in at least four different high-power fields (500 μm^2^) in two to three different lung sections per mouse by using Fiji ImageJ. Data represent mean ± SEM. *p < 0.05, **p < 0.01, and ***p < 0.001.

The AhR reporter plasmids PGL3-6xDRE (WT)-green fluorescent protein (GFP) and PGL3-6xDRE (mut)-GFP were generated as illustrated in Figure 1E. PGL3-6xDRE (WT)-GFP consists of the mouse CYP1A1 promoter sequences (0–1,400 bp) upstream of the GFP gene in pGL3-basic vectors (Promega). PGL3-6xDRE (mut)-GFP plasmid was generated by using the QuikChange® Site-Directed Mutagenesis Kit that contains mutations in dioxin-responsive element (DREs) of PGL3-6xDRE-GFP. Both AhR reporter plasmids were transfected into 16HBECs and cultured for 24 h. The 16HBECs containing the aryl hydrocarbon receptor DRE reporter plasmid were stimulated with different doses of BaP (0.01–10 μM) or Der f 1 (7.5–60 μg/ml) or combined BaP (1 μM) and Der f 1 (30 μg/ml) for 24 h and then lysed. The supernatants were harvested and plated in a 96-well plate for the quantification of GFP with the Multi-Mode Microplate Reader (BioTek, US). The results were normalized based on the negative GFP of the control.

### Plasmid Construction

The RhoA promoter construct was generated as previously described (42). Briefly, five constructs containing different truncated lengths of the RhoA promoter regulatory sequences were generated with mouse genomic DNA and the forward and reverse primers incorporating MluI and XhoI sites at the 5′ and 3′ ends, respectively. Both the amplified DNA products and pGL3-Basic Vector (Promega) were digested by MluI and XhoI enzymes and then linked by using ClonExpress II kit (Vazyme). The QuikChange® Site-Directed Mutagenesis Kit (Stratagene, La Jolla, CA) was used to generate the constructs for site-directed mutation. All of the above constructs were verified by sequencing. All the primers used are listed in Supplementary Table 2.

### Luciferase Reporter Assays

For luciferase reporter assays of promoter activity, 16HBECs were transfected with RhoA promoter plasmids and pGL3-basic (RiboBio) using Lipofectamine 2000 (Thermo Fisher Scientific) for 24 h and then treated with BaP (1 μM). After 24 h, these transfected and treated 16HBECs were lysed using RIPA buffer (Solarbio Beijing, China) and centrifuged. The supernatants were used for the measurement of luciferase activity in a Multi-Mode Microplate Reader (BioTek, US). The promoter activity was presented as fold change relative to the luciferase activity of the empty plasmid.

### Statistical Analysis

All data were analyzed with Graph Pad Prism version 5.1 software (GraphPad Software, La Jolla, CA) and are expressed as mean ± SEM. The statistical significance for normally distributed samples was assessed using an independent two-tailed Student's *t*-test or with one-way analysis of variance followed by Tukey's *post-hoc*-test. A *p*-value < 0.05 was considered statistically significant for all analyses. All experiments *in vitro* were performed two or more times independently under identical or similar conditions.

## Results

### BaP Co-Exposure Exacerbates Der f 1-Induced TGFβ1 Secretion and Signaling Activation

Airway epithelial cells are one of the major sources of TGFβ1, and activation of TGFβ1 signaling plays an important role in the allergen-induced AHR and airway inflammation in asthma (34, 36). Thus, we investigated whether BaP alone or co-exposure can induce or enhance TGFβ1 production in the airway epithelial cells. As expected, BaP exposure significantly induced TGFβ1 production in 16HBECs in a dose-dependent (Figure 1A) and time-dependent (Figure 1B) response. Furthermore, Der f 1 exposure can also induce TGFβ1 production, which was further potentiated by BaP co-exposure in 16HBECs (Figure 1C). To confirm the BaP co-exposure-induced TGFβ1 expression in the surface of airway epithelial cells, the expression of TGFβ1 was detected by immunofluorescent staining. Consistently, BaP co-exposure with Der f 1 induced an increased expression of TGFβ1 in 16HBECs (Figures 1D,E). The same pattern was also noted for phospho-SMAD3 (p-SMAD3) (Figures 1D,F), representing the activation of the SMAD signaling pathway downstream to TGFβ1 (43). In addition, we detected TGFβ1 in the airway epithelial cells of an asthma mouse model by co-immunofluorescent staining TGFβ1 with epithelial cell marker EpCAM. As expected, a significantly increased expression was observed for TGFβ1 in the airway epithelial cells of either BaP- or Der f 1-treated mice (Figures 1G,H), which was further enhanced in mice when exposed to both BaP and Der f 1. Of note is the fact that the same pattern was observed for p-Smad3 (Figures 1G,I). Collectively, these results suggest that BaP co-exposure can potentiate Der f 1-induced epithelial TGFβ1 release and signaling activation.

### Co-Exposure of Der f 1 With BaP Increases BaP-Induced AhR Activity

**Figure 2:**
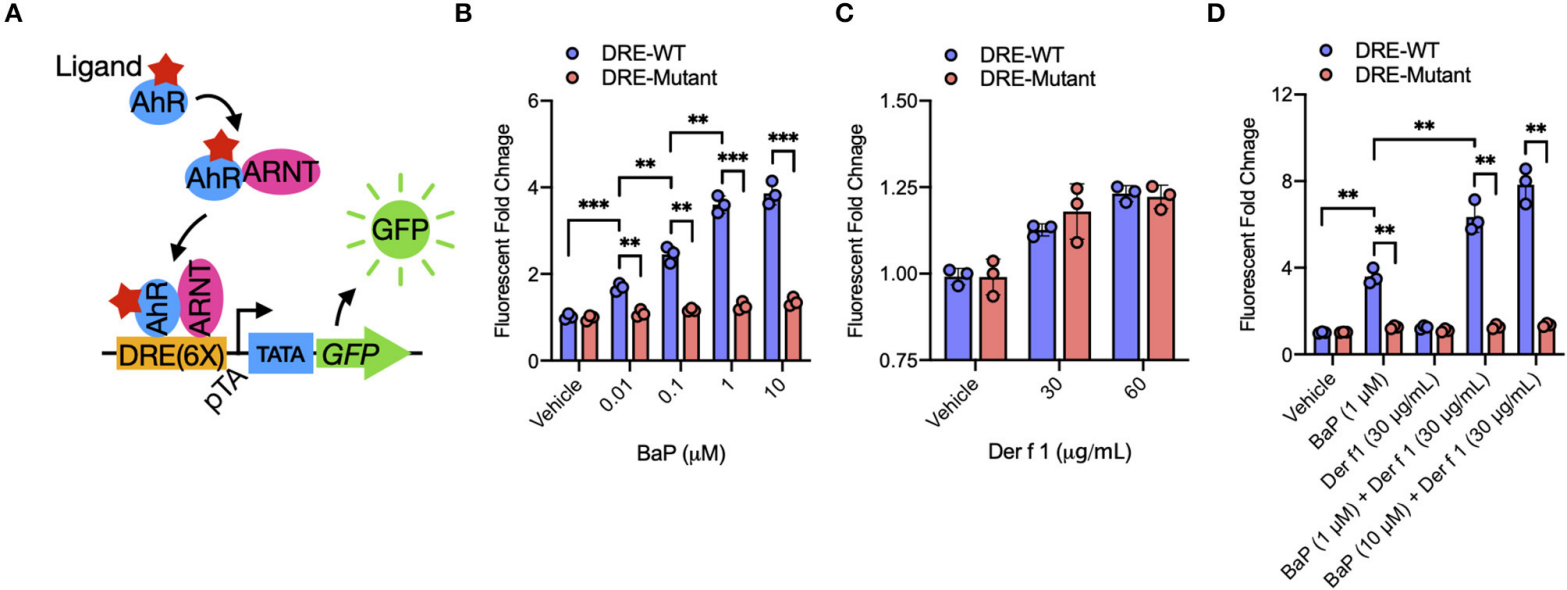
BaP co-exposure promotes Der f 1-induced AhR activity. **(A)** Schematic of experimental workflow for AhR reporter plasmid. Upon ligand binding, the AhR translocates into the nucleus and heterodimerizes with AhR nuclear translocator (ARNT). The AhR/ARNT complex binds to dioxin response elements in the upstream regulatory regions of the AhR target gene Cyp1a1 and induces gene activation. **(B–D)** Relative AhR activity in BaP **(B)**, Der f 1 **(C)**, and BaP + Der f 1-treated 16HBECs **(D)** that were transfected with AhR reporter plasmid. AhR activity was assessed by green fluorescent protein quantification and expressed as fold changes relative to the control. Data represent mean ± SEM. *p < 0.05, **p < 0.01, and ***p < 0.001.

BaP is one of the ligands to AhR (17). We have previously shown that BaP can induce the expression of AhR and its major downstream gene *CYP1A1* in 16HBECs (29). Here we extended to examine whether BaP exposure alone or co-exposure can directly induce the activation of AhR signaling by transfecting an AhR–DRE reporter plasmid (Figure 2A). The *GFP* gene is under control of tandem repeats of the DRE [PGL3-6xDRE (WT)-GFP, PGL3-6xDRE (mut)-GFP]. The PGL3-6xDRE (WT)-GFP or PGL3-6xDRE (mut)-GFP plasmid was transduced in 16HBECs, cultured for 24 h, and then treated with different doses of BaP (0.01–10 μM) and Der f 1 (0–60 μg/ml). BaP exposure induced AhR activation, as assessed by GFP quantification, in a dose-dependent manner (Figure 2B). By contrast, a dose-dependent AhR activation was not noted for Der f 1 exposure (Figure 2C). Of interest is that the cultures treated with both BaP and Der f 1 showed a significant increase in AhR activation relative to those treated with BaP alone (Figure 2D), indicating that the co-exposure of Der f 1 with BaP increases BaP-induced AhR activity in airway epithelial cells.

### AhR Mediates BaP Co-Exposure-Induced Epithelial TGFβ1 Secretion and Signaling Activation

**Figure 3:**
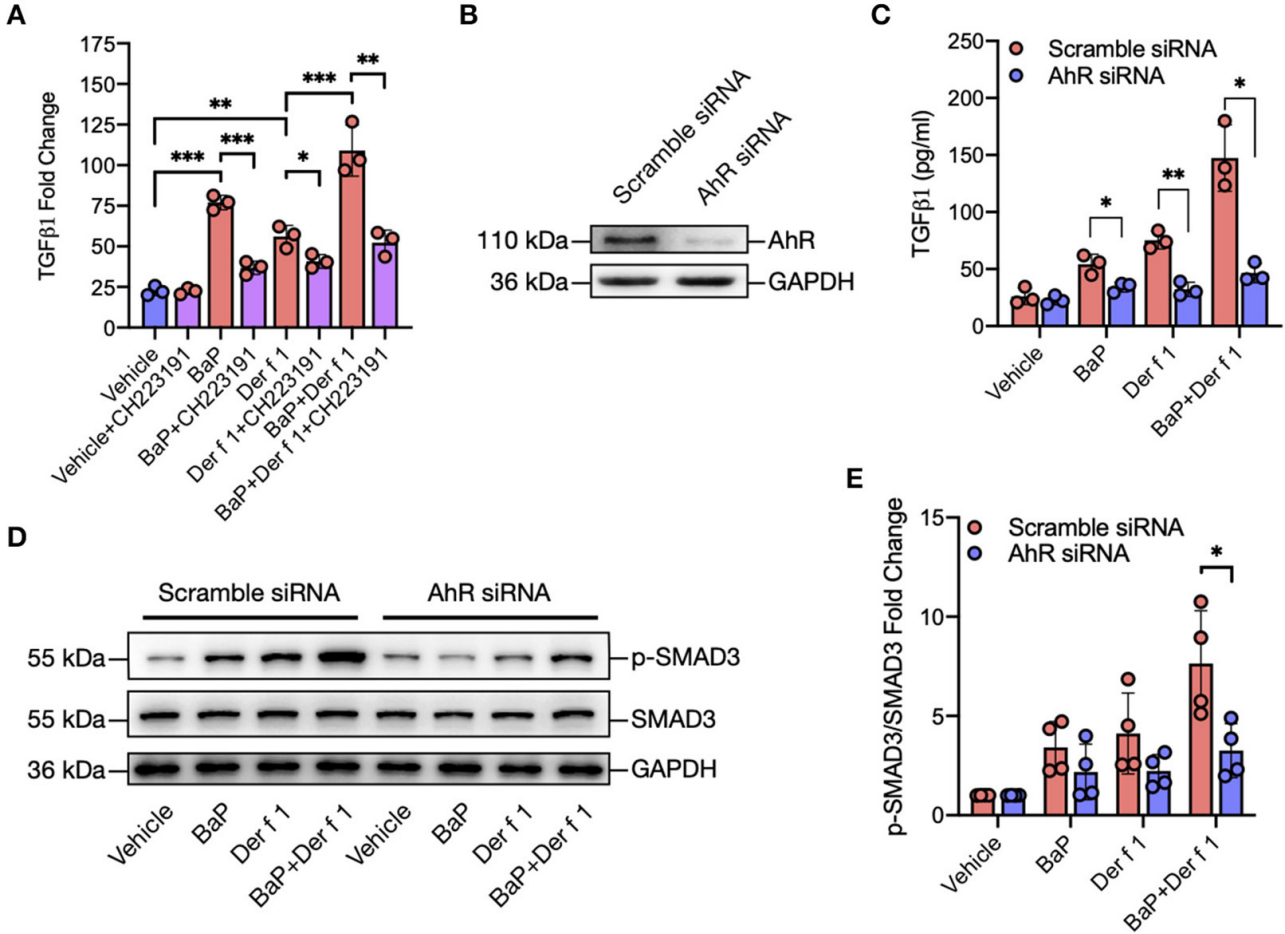
AhR mediates BaP co-exposure-induced TGFβ1 secretion and signaling activation. **(A)** Levels of TGFβ1 in supernatants of 16HBECs treated with BaP (1 μM) and Der f 1 (30 μg/ml) alone or in combination in the presence or absence of CH223191 (10 μM). **(B)** Western blot analysis of AhR knockdown in 16HBECs. **(C)** Levels of TGFβ1 in supernatants of 16HBECs with or without AhR knockdown after exposure to BaP (1 μM) and Der f 1 (30 μg/ml) alone or in combination. **(D)** Western blot analysis of p-Smad3 expression in BaP co-exposure-treated 16HBECs with or without AhR knockdown. **(E)** Quantitative analysis of Western blots in **(D)**. Data represent mean ± SEM. *p < 0.05, **p < 0.01, and ***p < 0.001.

We further investigated whether active AhR plays a role in TGFβ1 release from airway epithelial cells. 16HBECs were pre-treated with AhR antagonist CH223191 and then exposed to either BaP or Der f 1 or their combination for 24 h. Their levels of TGFβ1 in supernatants were measured. Of note is that BaP co-exposure-induced TGFβ1 production was almost completely blocked in CH223191 pre-treated 16HBECs (Figure 3A). To further understand the role of AhR in TGFβ1 secretion, AhR in 16HBECs was knocked down by siRNA and confirmed by Western blotting (Figure 3B). Consistent with the findings with AhR antagonist CH223191, the levels of TGFβ1 were reduced in AhR-deficient 16HBECs after exposure to BaP alone or BaP co-exposure with Der f 1 (Figure 3C). Furthermore, Western blotting illustrated that BaP alone or co-exposure induced the expression of phosphorylated Smad3 (p-SMAD3), which was reduced in 16HBECs with AhR knockdown (Figures 3D,E). These findings imply that AhR activation is essential in mediating BaP-induced airway epithelial TGFβ1 release and signaling activation.

### RhoA Mediates the Regulation of AhR on BaP Co-Exposure-Induced TGFβ1 Production

**Figure 4:**
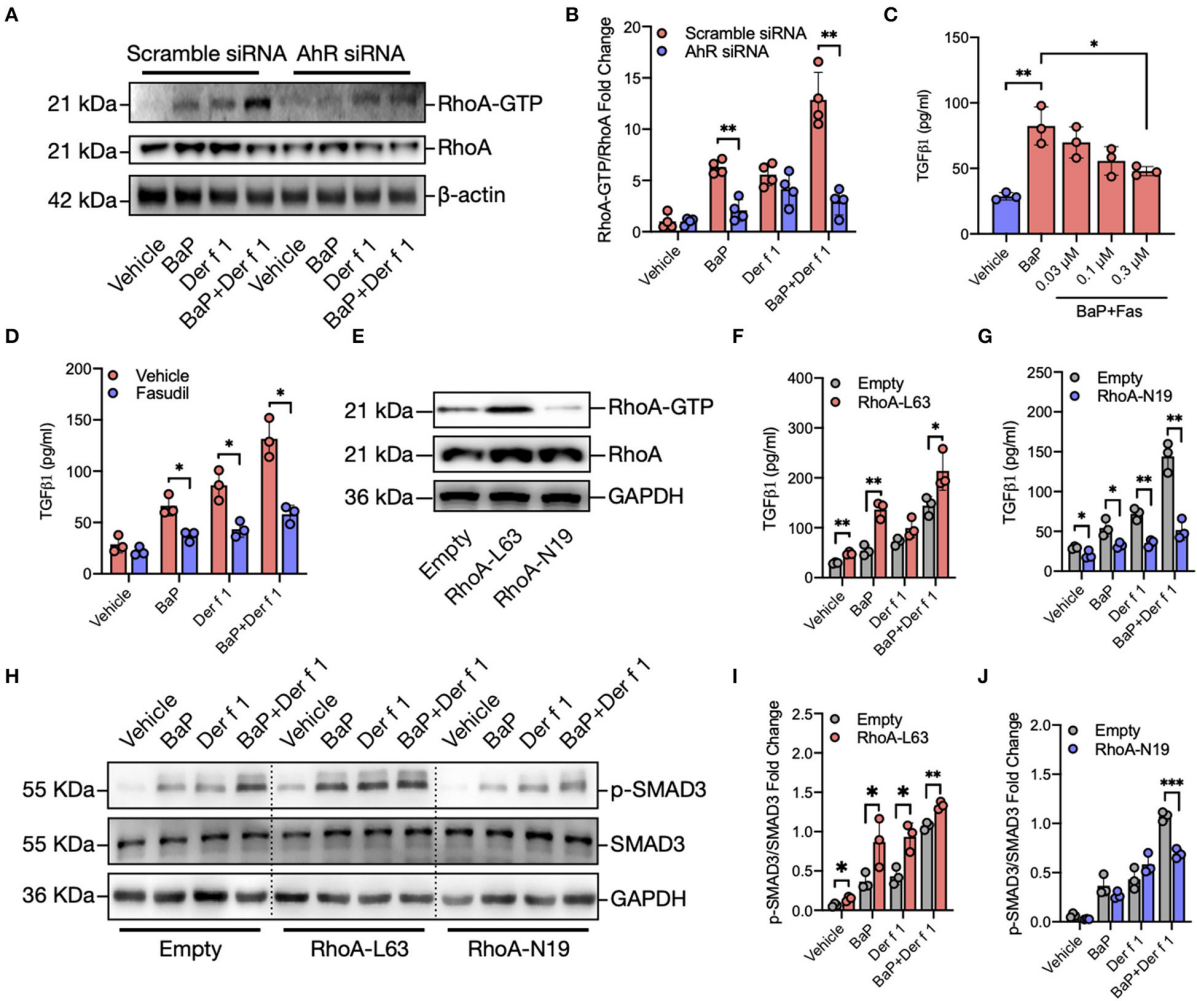
RhoA mediates the regulation of AhR on BaP co-exposure-induced TGFβ1 production and signaling activation. **(A)** Western blot analysis of RhoA-GTP expression in BaP (1 μM) and Der f 1 (30 μg/ml) or combination-treated 16HBECs with or without AhR knockdown. **(B)** Quantitative analysis of Western blots in **(A)**. **(C)** Levels of TGFβ1 in supernatants of BaP-treated 16HBECs with or without fasudil at varying doses (0.03–0.3 μM). **(D)** Levels of TGFβ1 in supernatants of BaP co-exposure-treated 16HBECs in the presence or absence of fasudil (0.3 μM). **(E)** Western blot analysis of RhoA-GTP expression in 16HBECs expressing either a constitutively active RhoA (RhoA-L63) or dominant-negative RhoA (RhoA-N19). **(F,G)** Levels of TGFβ1 in supernatants of BaP (1 μM), Der f 1 (30 μg/ml), and co-exposure-treated 16HBECs expressing either RhoA-L63 **(F)** or RhoA-N19 **(G)**. **(H–J)** Western blot analysis of p-Smad3 expression in BaP (1 μM), Der f 1 (30 μg/ml), and co-exposure-treated 16HBECs expressing either RhoA-L63 **(H,I)** or RhoA-N19 **(H,J)**. Data represent mean ± SEM. *p < 0.05, **p < 0.01, and ***p < 0.001.

To explore the underlying mechanisms for how AhR activation regulates TGFβ1 secretion and signaling activation, we specifically investigated whether RhoA/Rho-kinase activation plays a role in BaP co-exposure-induced epithelial TGFβ1 release and signaling activation. Previous studies have suggested a feed-forward connection between RhoA/Rho-kinase and TGFβ1 (38–41), raising the possibility that RhoA/Rho-kinase may function as a central player in connecting the upstream BaP-induced AhR activation with the downstream TGFβ1 production. To test whether AhR, upstream to RhoA, is involved in regulating BaP co-exposure-induced active RhoA, we performed Western blot analysis to detect the expression of active RhoA in16 HBECs with or without AhR knockdown. An increased expression of active RhoA (RhoA-GTP) was detected in 16HBECs after exposure to either BaP (1 μM) or Der f 1 (30 μg/ml) alone or in combination (Figures 4A,B). Of note is that the increased expression of RhoA-GTP was reduced in 16HBECs with AhR knockdown, particularly in BaP or co-exposure-treated 16HBECs, suggesting that AhR regulates BaP or co exposure-induced RhoA/Rho-kinase activation in 16HBECs. Next, we examined the role of active RhoA in BaP co-exposure-induced downstream TGFβ1 production by using fasudil, a selective RhoA/ROCK inhibitor (44). Pre-treatment of 16HBECs with fasudil showed a dose-dependent inhibition in BaP-induced TGFβ1 secretion (Figure 4C). The inhibition was observed not only for BaP but also for Der f 1 or BaP co-exposure-induced TGFβ1 secretion (Figure 4D). To further confirm the role of active RhoA in regulating TGFβ1 secretion and signaling activation, we used 16HBECs expressing either a constitutively active RhoA (RhoA-L63) or dominant-negative RhoA (RhoA-N19) (Figure 4E). Compared to 16HBECs, 16HBECs expressing RhoA-L63 secreted higher levels of TGFβ1 in response to BaP alone or BaP co-exposure with Der f 1 (Figure 4F). In contrast to the results, a significant reduction was noted for TGFβ1 secretion in 16HBECs expressing RhoA-N19 (Figure 4G). The same patterns were observed for p-SMAD3 in 16HBECs expressing RhoA-L63 or RhoA-N19 (Figures 4H,I). Collectively, these results suggest that RhoA may play an important role in mediating the regulation of AhR on BaP co-exposure-induced TGFβ1 production and signaling activation.

### AhR Directly Binds to the Promoter Region of RhoA and Participates in the BaP-Induced RhoA Activity

**Figure 5:**
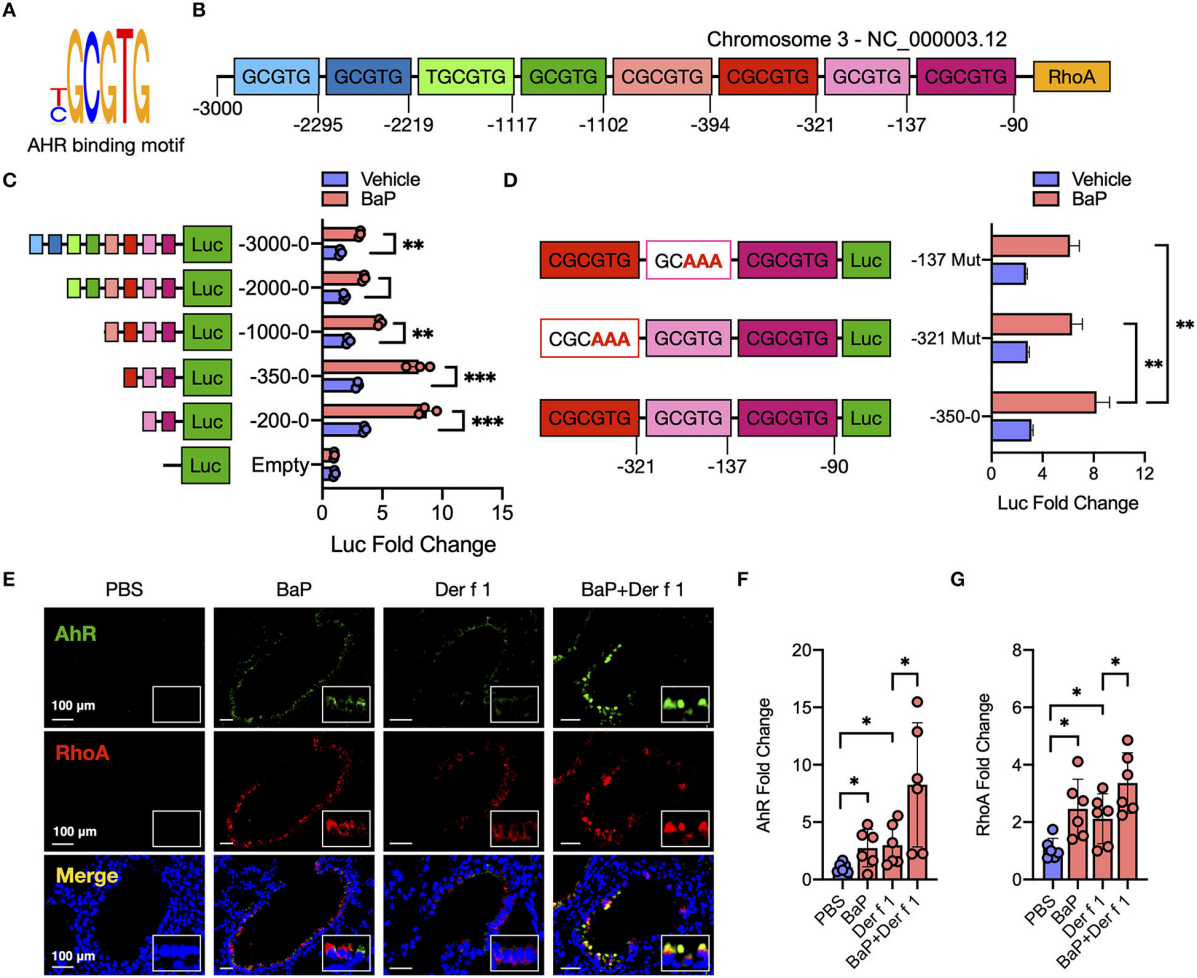
AhR directly binds RhoA and participates in the BaP-induced RhoA activity. **(A)** Sequence of AhR binding site. **(B)** Eight putative AhR binding sites in the promoter region of RhoA. **(C)** Luciferase reporter assays with luciferase reporter vector pGL-3 basic (empty) or containing serially truncated fragments of the RhoA promoter (AhR reporter) in 16HBECs. **(D)** Luciferase reporter assays with luciferase reporter vector pGL-3 basic containing wild-type or mutated truncated fragments in 16HBECs. **(E)** Representative immunofluorescence images of AhR, RhoA, and co-localized expression in the lung tissues of a mouse model. **(F,G)** Quantitative analysis of the fluorescent signal in **(E)** (*n* = 6 mice/group). Fluorescent-positive cells of tissue sections for AhR **(F)** and RhoA **(G)** were quantified in at least four different high-power fields (500 μm^2^) in two to three different lung sections per mouse by using Fiji ImageJ. Data represent mean ± SEM. *p < 0.05, **p < 0.01, and ***p < 0.001.

To determine whether AhR regulates RhoA activation through direct interaction with RhoA, we searched for the AhR binding sites (T/CGCGTG, Figure 5A) in the promoter region of RhoA by using *in silico* analysis with BiBiServ RNAhybrid, a program that predicts multiple potential binding sites (45). Eight putative AhR binding sites that may affect transcriptional activation were predicted (Figure 5B). To confirm the significance of these binding sites, we performed luciferase reporter assays with luciferase reporter vector pGL-3 basic with various truncated fragments of the RhoA promoter containing AhR binding sites (Figure 5C). A total of five different truncated lengths of the RhoA promoter regulatory sequences were amplified, and the PCR products were cloned into the pGL3-basic vector. All fragments showed a better response to BaP as compared to those with pGL3-basic vector. Of these, fragments containing AhR binding sites 6–8 and site 7 and 8 showed a prominently increased promoter activity. To confirm the significance of binding sites in regulating the promoter activity of RhoA, we mutated binding site 6 or 7 and examined their effects on promoter activity. Compared to the wild type, the fragment containing mutated binding site 6 or site 7 showed a significantly reduced promoter activity (Figure 5D). Furthermore, we examined the co-localization between AhR and RhoA in the lung tissues of an asthma mouse model (Figure 5E) and found a clear overlapping between AhR and RhoA-GTP in the airways of either BaP or Der f 1 alone or co-exposure-treated mice, supporting that there might be an interaction between AhR and RhoA. Similar to the previous findings, BaP co-exposure-treated mice showed an increased expression of AhR (Figures 5E,F) and active RhoA (Figures 5E,G) compared to BaP or Der f 1 alone. Collectively, these findings indicate that AhR may regulate BaP-induced RhoA activity in the airways *via* directly binding the promoter region of RhoA.

### Inhibition of RhoA Suppresses BaP Co-Exposure-Induced AHR and Airway Inflammation

**Figure 6:**
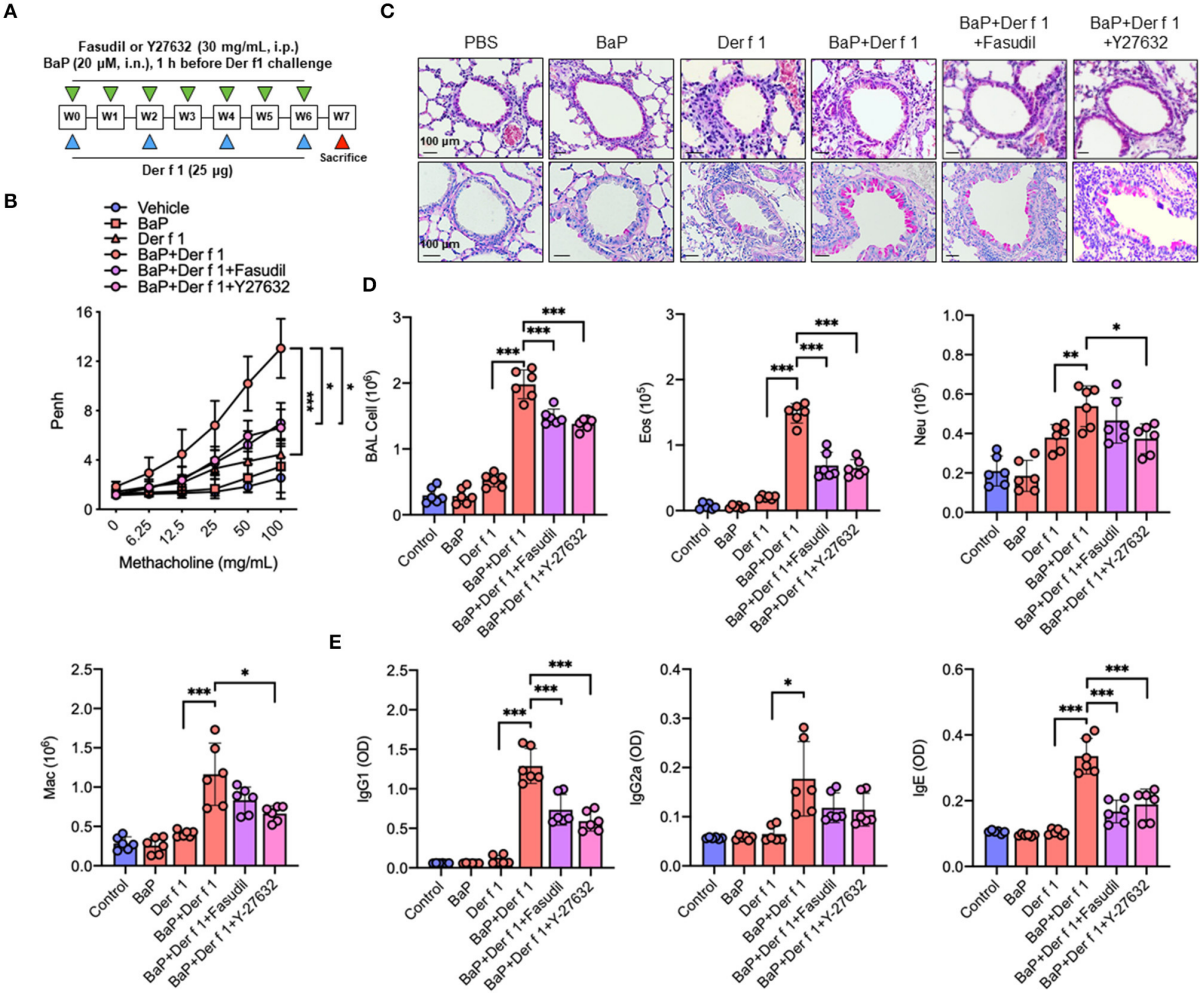
Inhibition of RhoA suppresses BaP co-exposure-induced AHR and Th2-associated airway inflammation. **(A)** Protocol for BaP (20 μM) and Der f 1 (25 μg) co-exposure-induced mouse model of asthma in the presence or absence of Fasudil or Y-27632 (30 mg/ml, i.p.). **(B)** Lung resistance in response to increasing concentrations of methacholine using the forced oscillation technique (FlexiVent, SCIREQ). **(C)** Histological examination of mouse paraffin lung sections stained with hematoxylin and eosin (upper panel) and periodic acid–Schiff (lower panel). **(D)** Bronchoalveolar lavage fluid total and differential (eosinophil, neutrophil, and macrophage) cell count. **(E)** Serum levels of house dust mite-specific IgG1, IgG2a, and IgE. Data represent mean ± SEM (*n* = 6/group). *p < 0.05, **p < 0.01, and ***p < 0.001.

Given that AhR can regulate RhoA activation, we examined whether RhoA plays a role in BaP co-exposure-induced AHR and airway inflammation by using RhoA inhibitors fasudil and Y-27632 in our mouse model of asthma as illustrated in Figure 6A. BaP and Der f 1 co-exposure induced an increased airway resistance when compared with either BaP or Der f 1 alone. Of note is that the increased airway resistance was significantly inhibited by either fasudil or Y-27632 treatment (Figure 6B). Consistently, histological analysis demonstrated that BaP co-exposure increased airway inflammation as assessed by peribronchial inflammation (H&E, upper panel) and goblet cell hyperplasia (PAS, lower panel) (Figure 6C). Of interest is that the increased lung inflammation was significantly abrogated when these BaP co-exposure-challenged mice were pre-treated with either fasudil or Y-27632 treatment. The same pattern was also observed for total cell counts in BAL samples, particularly eosinophils and macrophages (Figure 6D), and serum levels of Der f 1-specific IgE, IgG1, and IgG2a (Figure 6E). The results suggest that inhibition of RhoA suppresses Bap co-exposure-induced AHR and airway inflammation.

### Inhibition of RhoA Suppresses BaP Co-Exposure-Induced Cytokine Release

**Figure 7:**
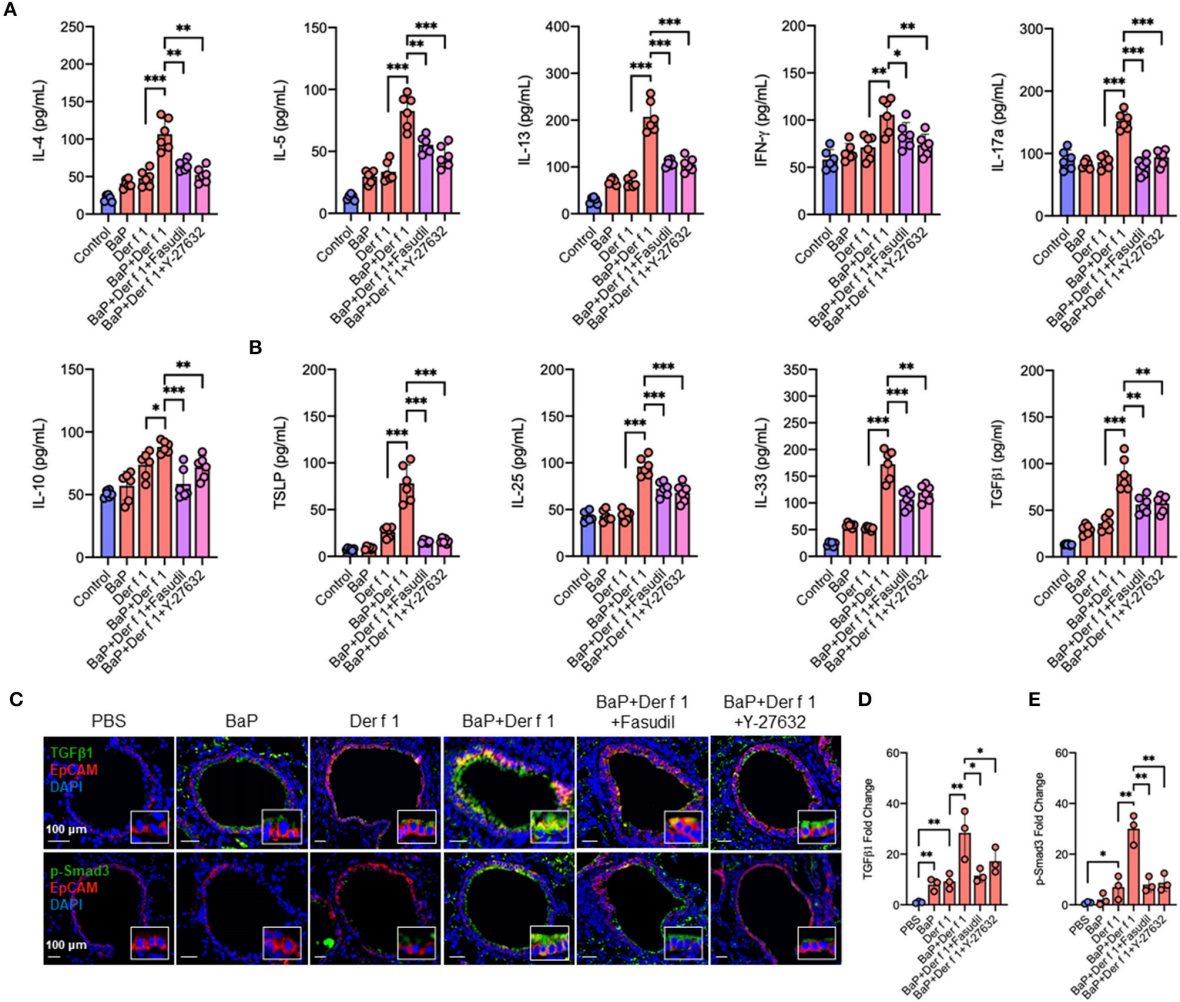
Inhibition of RhoA suppresses BaP co-exposure-induced cytokine production. **(A,B)** Levels of Th1/Th2/Th17 inflammatory cytokines **(A)** and airway epithelial cell-derived cytokines **(B)** in the bronchoalveolar lavage samples of mice treated with either Fasudil or Y-27632. **(C)** Representative immunofluorescence images of TGFβ1 (green, upper panel) and p-Smad3 (green, lower panel) expression in the airway epithelial cells (EpCAM, red) of an asthma mouse model. **(D,E)** Quantitative analysis of fluorescent-positive cells in the tissue sections for TGFβ1 **(D)** and p-Smad3 **(E)** in at least four different high-power fields (500 μm^2^) in two to three different lung sections per mouse by using Fiji ImageJ. Data represent mean ± SEM (*n* = 6 mice/group). *p < 0.05, **p < 0.01, and ***p < 0.001.

We have specifically investigated whether RhoA participates in the regulation of cytokine production. As compared to Der f 1 alone, we found that BaP co-exposure enhanced the production of IL-4, IL-5, IL13, IFNγ, IL-17, and IL-10 in BAL samples. As expected, the increased cytokine release was remarkably inhibited in mice pre-treated with either fasudil or Y-27632 as compared to the vehicle control (Figure 7A). Furthermore, we detected whether inhibition of RhoA can affect BaP co-exposure-induced epithelial cytokine release. We found higher levels of IL-25, IL-33, TSLP, and TGFβ1 in BAL samples of BaP co-exposure-treated mice as compared to those pre-treated with Der f 1. Of note is that all of these increased cytokines were remarkably inhibited by either fasudil or Y-27632 (Figure 7B), indicating that inhibition of RhoA suppresses BaP co-exposure-induced airway epithelial-derived cytokine release.

### Inhibition of RhoA Suppresses BaP Co-Exposure-Induced TGFβ1 Expression and Signaling Activation in a Mouse Model of Asthma

We further confirmed the role of RhoA in TGFβ expression and signaling activation in the airway epithelium of an asthma mouse model by co-immunostaining with epithelial marker EpCAM. Notably, BaP and Der f 1 co-exposure enhanced TGFβ1 expression in airway epithelial cells (Figures 7C,D). The increased expression was significantly reduced in those treated with either fasudil or Y-27632. A similar pattern was also observed for p-Smad3 (Figures 7C,E). BaP co-exposure promoted p-Smad3 expression, which was inhibited by either fasudil or Y-27632 treatment. The data together provide a mechanistic explanation for the role of AhR-regulated RhoA activation in epithelial cell TGFβ1 release, signaling activation, and subsequently Th2-associated allergic airway inflammation.

## Discussion

Airway epithelium lining the upper and lower respiratory tract forms the first line of defense of the airway and lungs against inhaled pathogens and environmental pollutants (46–48). We and others have previously demonstrated that environmental pollutants can promote the allergen-induced release of ROS and epithelial-derived cytokines (e.g., IL-25, IL-33, and TSLP) that can enhance DC maturation and type 2 innate lymphoid cells and Th2 responses (29, 49, 50). Furthermore, AhR, as a dioxin receptor and environmental sensor, has been suggested to mediate DEP component-induced oxidative stress, epithelial cytokine release, Th17 immune responses, and allergic airway inflammation (29, 51). In the current study, we made a novel finding that BaP co-exposure can enhance Der f 1-induced TGFβ1 release and signaling activation that may contribute to the exacerbation of allergic airway inflammation. Most importantly, we identified a functional axis of AhR–RhoA that regulates epithelial TGFβ1 release and signaling activation.

TGFβ1 regulates fundamental cell biological functions, including cell growth, differentiation, migration, and activation (30–35). There is increasing evidence to demonstrate that TGFβ1 is one of the major immune-regulatory cytokines from airway epithelial cells that recruit various immune cells to the airways and interact with them to regulate AHR and airway inflammation in asthma (34, 36). On one hand, TGFβ1 can modulate lineage differentiation of T cells into different effector T cell subsets (52). On the other hand, TGFβ1 is a cofactor of innate lymphoid cells to induce type-2 airway inflammation and key features of allergic diseases (34, 53). Thus, it is pivotal to determine how the increased TGFβ1 production is regulated, which offers novel insights into the mechanisms that lead to allergic airway inflammation. Here we demonstrated that TGFβ1 was increased not only in supernatants (soluble TGFβ1) of BaP/Der f 1-treated human airway epithelial cells but also in airway epithelial cells (membrane-bound form) of a BaP/Der f 1-treated mouse model of asthma. Soluble TGFβ1 has been the major focus of previous investigations, but the functional membrane-bound TGFβ1 is equally important. While any differences for soluble and cell-specific membrane-bound TGFβ1 in their downstream functions are not clear, our data generated from this study support our hypothesis that BaP co-exposure can exacerbate Der f 1-induced TGFβ1 expression in both soluble and membrane-bound form. Furthermore, the increased expression was also observed for p-Smad3, a downstream molecule of canonical TGFβ1 signaling, indicating that BaP co-exposure can potentiate Der f 1-induced epithelial TGFβ1secretion and signaling pathway activation.

Next, we explored how AhR regulates TGFβ1 activation and signaling pathways. AhR as a ligand-activated transcription factor senses and responds to environmental stimuli and regulates normal cell development and innate and adaptive immune responses (19–27). Our previous studies have suggested that AhR controls mast cell growth and activation (54), cockroach allergen-induced immune responses in human fibroblast (55), and allergic airway inflammation in a mouse model of asthma (35). Furthermore, we found that BaP exposure can activate AhR that subsequently leads to a promotion of Der f 1-induced oxidative stress and IL-25, IL-33, and TSLP release from the epithelium (29). Our findings were supported by some other studies showing that BaP can directly induce AhR activation that contributes to the pro-inflammatory response in respiratory allergy through enhancing IL-33 expression and eosinophil infiltration (17). In the current study, we extended to detect the BaP co-exposure-induced activation of AhR signaling by using AhR reporter plasmid (PGL3-6xDRE-GFP). The plasmid was specifically designed to see whether the ligand-induced AhR can translocate into the nucleus and bind to the upstream regulatory regions of Cyp1a1 with DREs and induce gene activation as assessed by GFP quantification (56). GFP is only expressed when AhR ligands bind to DRE. Intriguingly, we noted that BaP co-exposure showed an increased activation of AhR, suggesting a synergistic effect between BaP and Der f 1. However, the underlying mechanisms as for how both BaP and Der f 1 induce increased AhR activation remain to be determined. Furthermore, we illustrated that AhR activated by BaP modulates TGFβ1 release and p-Smad3 expression in airway epithelial cells. Collectively, our findings provided evidence that active AhR is critical in controlling the activation of the TGFβ1/Smad3 signaling axis in airway epithelial cells.

**Figure 8:**
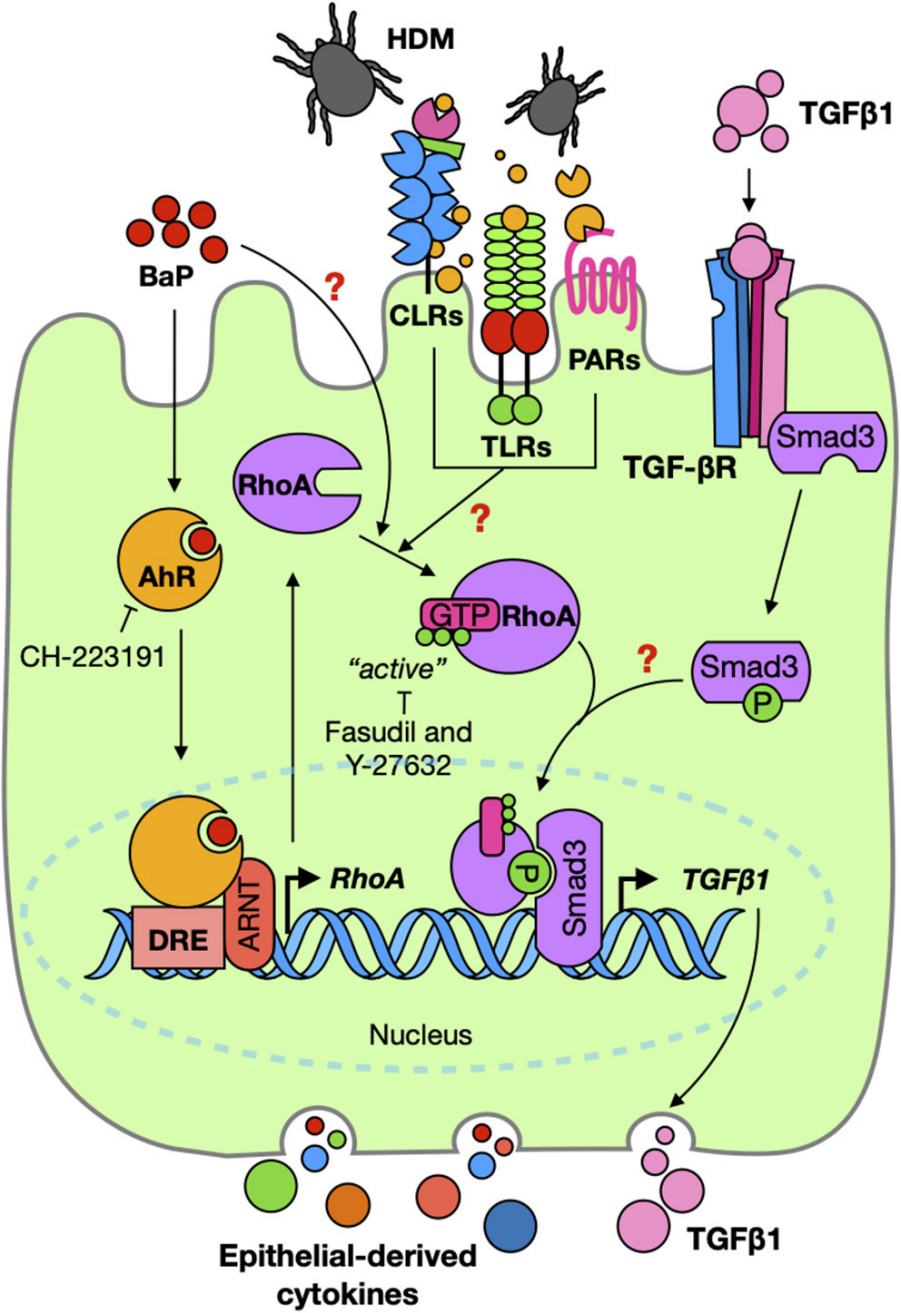
Graphic summary. BaP co-exposure exacerbates Der f 1-induced activation of TGFβ1/Smad3 signaling through the AhR–RhoA axis in epithelial cells.

To further address the relative contribution of AhR in the activation of the TGFβ1/Smad3 signaling axis, we studied its underlying mechanisms by focusing on RhoA/Rho-kinase signaling. RhoA is a nucleotide-dependent protein, switching between an inactive form, GDP-bound RhoA, and an active form, GTP-bound RhoA, and has been shown to regulate various biological functions, including cell differentiation, differentiation, recruitment, and apoptosis (37, 57, 58). Increased expression and activation of RhoA have been associated with asthma (59–62). Importantly, several studies including ours have suggested a feed-forward circuit between RhoA signaling and TGFβ1 that drives airway constriction, airway hyper-responsiveness, and airway remodeling in asthma (38–41). Thus, it is possible that RhoA/Rho-kinase signaling may function as a central player in connecting the upstream BaP-induced AhR activation with the downstream TGFβ1 production and signaling activation. This was supported by our observations that BaP induces active RhoA presented in epithelial cells as determined by the expression of RhoA-GTP (active RhoA). Particularly, we showed that AhR, as a receptor for BaP, is involved in regulating BaP-induced RhoA activation. Furthermore, we found a co-localization between AhR and RhoA-GTP in the airways of an asthma mouse model, indicating a possible interaction between AhR and RhoA-GTP. However, the underlying mechanisms by which AhR regulates RhoA activation remain unknown. We postulated that AhR regulates RhoA activation through binding to the promoter region of RhoA and inducing RhoA expression, which amplified the allergen or TGF-β1 or others-induced RhoA activation (Figure 8). As expected, eight putative AhR binding sites in the promoter region of RhoA were predicted by using *in silico* analysis. These AhR binding sites were validated by luciferase reporter assays with different truncated fragments of the RhoA promoter. In contrast to those containing the mutated binding sites, all fragments containing the putative AhR binding sites in the promoter region of RhoA showed an increased promoter activity in response to BaP treatment. Although our data suggest a functional axis of AhR–RhoA, the detailed mechanisms as to how AhR regulates RhoA activation remain to be further investigated. In addition, we demonstrated for the first time that Der f 1 alone could induce the activation of RhoA/Rho-kinase. While the reason is unknown, further studies would be of interest to determine whether Der f 1 either directly activates RhoA or induce RhoA activation through its possible receptor PAR2, C-type lectin receptors, or Toll-like receptors (Figure 8).

We also provided evidence that active RhoA/Rho-kinase and its signaling are critical in BaP-induced TGFβ1 production and signaling activation. 16HBECs expressing constitutively active RhoA-L63 secreted higher levels of TGFβ1 and an increased expression of p-Smad3 in response to BaP alone or BaP co-exposure with Der f 1. Furthermore, RhoA/ROCK inhibitor fasudil significantly inhibited BaP co-exposure-induced TGFβ1 secretion and p-Smad3 expression in airway epithelial cells. These findings suggest that active RhoA/Rho-kinase and its signaling modulate BaP co-exposure-induced TGFβ1 production and signaling activation. Additionally, it is well-recognized that TGFβ1 initiates their cellular functions by binding to the cell surface TGFβ receptor complex, which triggers activation of intracellular signaling molecule Smad2/3 in the cytoplasm (43, 63). Furthermore, the increased TGFβ1 signaling activation (e.g., p-Smad3) may, in turn, promote RhoA activation according to the previous findings (38–41). Collectively, these findings imply that BaP promotes RhoA activation through AhR, thereby leading to the production of TGFβ1 and the activation of TGFβ1 signaling. Furthermore, there may be a feed-forward connection between RhoA activation and TGFβ1 signaling activation (Figure 8).

Previous studies have demonstrated that RhoA/Rho-kinase plays an important role in regulating airway inflammation that may be through affecting the differentiation, recruitment, and activation of various inflammatory cells (e.g., eosinophils, macrophages, mast cells, and T cells) in the pathogenesis of asthma. Of these, RhoA was shown to control Th17 cell differentiation and house dust mite-induced allergic airway inflammation (61). Our findings in this study suggested the significance of active RhoA in the activation of the TGFβ1/Smad3 signaling axis. Our further study explored the potential in therapeutically translating these findings into the treatment of allergic asthma. We specifically investigated the role of RhoA in BaP co-exposure-induced AHR and allergic airway inflammation in asthma by using RhoA inhibitors Y-27632 and fasudil. Y-27632 is a primary and typical Rho-associated kinase inhibitor, but fasudil is another Rho-associated kinase inhibitor that has been approved for clinical use as an alternative to Y-27632 (64). In this study, we used both inhibitors to cross-validate the role of RhoA signaling in allergic airway inflammation. Most of the measures displayed similar effects or similar trends for both inhibitors. However, we found that a few measures showed effectiveness only for one of the RhoA inhibitors, suggesting a difference in effectiveness between the two inhibitors. Additionally, we found that inhibition of RhoA/Rho-kinase signaling suppresses the BaP co-exposure-induced increased TGFβ1 and p-Smad3 expression in lung tissues. These results together provide a mechanistic explanation for the role of RhoA/Rho-kinase signaling in AHR, Th2-associated allergic airway inflammation, and activation of the TGFβ1/Smad3 signaling pathway.

In summary, we have demonstrated that BaP co-exposure exacerbates Der f 1-induced activation of the TGFβ1/Smad3 signaling axis in airway epithelial cells. Mechanistically, AhR is critical in controlling the BaP co-exposure-induced activation of the TGFβ1/Smad3 signaling axis. Furthermore, we provided novel evidence that active RhoA/Rho-kinase plays an important role in connecting the upstream BaP-induced AhR activation with the downstream TGFβ1 production and signaling activation, and there is a feed-forward connection between RhoA activation and TGFβ1 signaling activation. Our further study revealed that inhibition of RhoA/Rho-kinase signaling significantly suppressed BaP co-exposure-induced AHR, Th2-associated airway inflammation, and airway epithelial cytokine release in a mouse model of asthma (Figure 8). Collectively, our studies identified a functional axis of AhR–RhoA that regulates the activation of TGFβ1/Smad3 signaling, thereby leading to the development of allergic airway inflammation and asthma. Blockage of the AhR–RhoA axis represents a promising novel therapeutic approach for the treatment of allergic asthma.

## Data Availability Statement

The original contributions presented in the study are included in the article/Supplementary Material, further inquiries can be directed to the corresponding authors.

## Ethics Statement

The animal study was reviewed and approved by The Animal Care and Use Committee in Peking University Shenzhen Graduate School.

## Author Contributions

EW, WT, DD, and XX performed the experiments, analyzed the data, and reviewed the manuscript. EW and PG wrote the manuscript. S-KH, ZL, and PG designed and supervised the study and wrote the manuscript. LY, XS, DX, and PY provided intellectual input and aided in the experimental design. All the authors read and approved the final version of the manuscript.

## Conflict of Interest

The authors declare that the research was conducted in the absence of any commercial or financial relationships that could be construed as a potential conflict of interest.

## Abbreviations

HDM: house dust mite
BaP: benzo(a)pyrene
Der f 1: dermatophagoides farina group 1
16HBEC: human bronchial epithelial cell
DEP: diesel exhaust particulates
AhR: aryl hydrocarbon receptor
RhoA: Ras homolog family member A
BALF: bronchoalveolar lavage fluid
AHR: airway hyper-responsiveness
DRE: dioxin-responsive element.
