# Supplementary material for: Benzo(a)pyrene Enhanced Dermatophagoides Group 1 (Der f 1)-Induced TGFβ1 Signaling Activation Through the Aryl Hydrocarbon Receptor–RhoA Axis in Asthma
Source: Front Immunol. 2021 Apr 15;12:643260. doi: 10.3389/fimmu.2021.643260 (PMC8081905; doi:10.3389/fimmu.2021.643260)
Supplement: Supplementary file 1 [file Data_Sheet_1.PDF]

TABLE E1. Antibodies used for western blot and immunofluorescence

Antibody	Clone	Species	Application	Source
Epi-CAM	G8.8	Rat	IF (1:100)	ThermoFisher
RhoA	EPR18134	Rat	WB (1:1000)	Abcam
RhoA-GTPase	26904	Mouse	IF (1:50)<br>WB (1:500)	New East<br>Bioscience
AhR	Polyclonal<br>Antibody	Rabbit	IF (1:50)<br>WB (1:1000)	Abcam
p-Smad3	EP823Y	Rabbit	IF (1:50)<br>WB (1:1000)	Abcam
Smad3	EP568Y	Rabbit	WB (1:1000)	Abcam
TGFβ1	EPR12078(B)	Rabbit	IF (1:50)	Abcam
GAPDH	D16H11	Rabbit	WB (1:2000)	CST
β-actin	8H10D10	mouse	WB (1:2000)	CST

GAPDH*, IF, Immunofluorescence; WB, Western blotting*

TABLE E2. Primers used for RhoA luciferase reporter assay

**Primers**	**Sequencing**
RhoA-F1	CGAGCTCTTACGCGTGCTAGCgagtagcagaacccagtgtag
RhoA-F2	CGAGCTCTTACGCGTGCTAGCagatcagaccacagccttgc
RhoA-F3	CGAGCTCTTACGCGTGCTAGCctggtgagggtcctaagggac
RhoA-F4	CGAGCTCTTACGCGTGCTAGCcgttagtgcgcacgcgtaaac
RhoA-F5	CGAGCTCTTACGCGTGCTAGCtgcttgcttaagggatgagtg
RhoA-R1	CTTACTTAGATCGCAGATCTCGAGgagggtagcgcgagagagc
R4-Mut-F	gtggggcctacttcgcgaacaagaagagttggcagttc
R4-Mut-R	gaactgccaactcttcttgttcgcgaagtaggccccac
R5-Mut-F	ggttttgcttttagggcaaagacgggctcctgagc
R5-Mut-R	gctcaggagcccgtctttgccctaaaagcaaaacc
