# Supplementary figures and images for: Benzo(a)pyrene Enhanced Dermatophagoides Group 1 (Der f 1)-Induced TGFβ1 Signaling Activation Through the Aryl Hydrocarbon Receptor–RhoA Axis in Asthma
Source: Front Immunol. 2021 Apr 15;12:643260. doi: 10.3389/fimmu.2021.643260 (PMC8081905; doi:10.3389/fimmu.2021.643260)

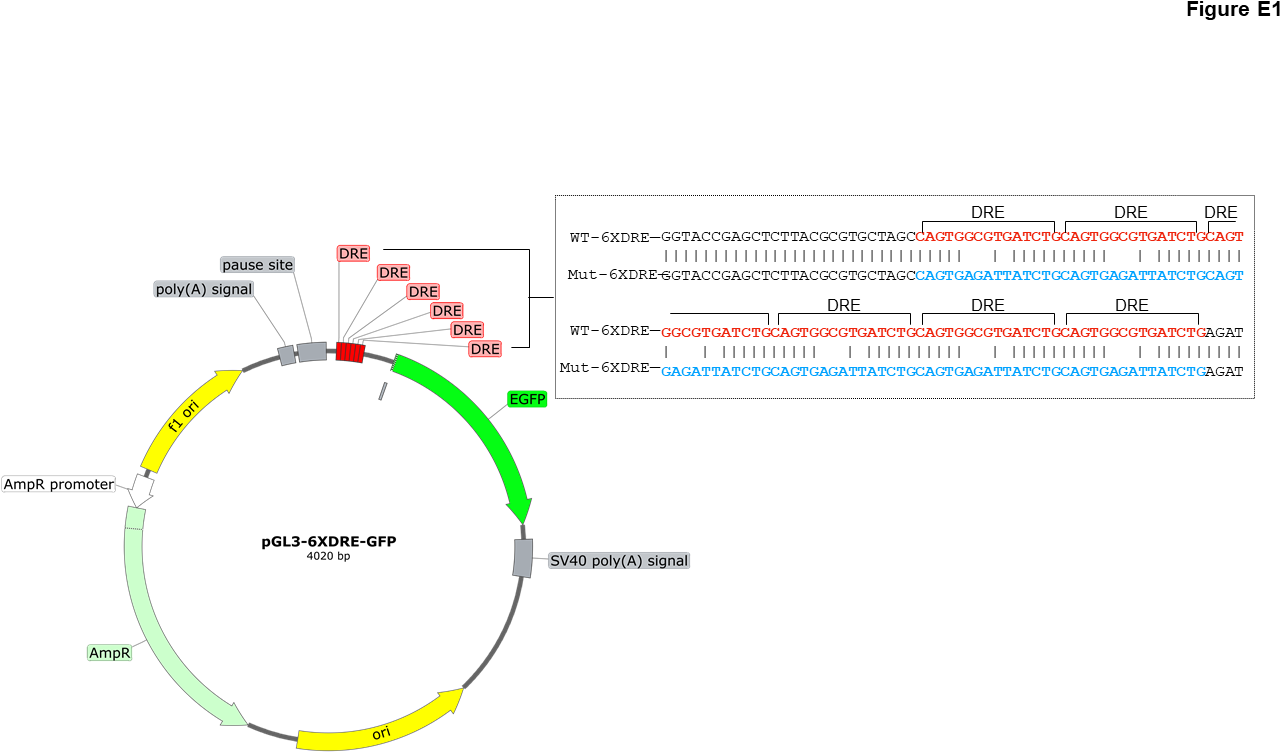

Supplement: Supplementary file 2 [file Image_1.TIF]
